# Comparison of Behavior and Genetic Structure in Populations of Family and Kenneled Beagles

**DOI:** 10.3389/fvets.2020.00183

**Published:** 2020-04-15

**Authors:** Borbála Turcsán, Kitti Tátrai, Eszter Petró, József Topál, Lajos Balogh, Balázs Egyed, Eniko Kubinyi

**Affiliations:** ^1^Department of Ethology, ELTE Eötvös Loránd University, Budapest, Hungary; ^2^Research Centre for Natural Sciences, Institute of Cognitive Neuroscience and Psychology, Hungarian Academy of Sciences, Budapest, Hungary; ^3^Department of Genetics, ELTE Eötvös Loránd University, Budapest, Hungary; ^4^Frédéric Joliot-Curie National Research Institute for Radiobiology and Radiohygiene, Budapest, Hungary

**Keywords:** kenneled dogs, family dogs, behavior test, canine microsatellites, population structure

## Abstract

In dogs, the social and spatial restriction associated with living in a kennel environment could lead to chronic stress and the development of abnormal behaviors (“kennel-dog syndrome”). However, little is known about how kenneled dogs differ from their conspecifics living as pets in human families. In the current study, using a test battery exposing the dogs to novel stimuli, we compared the behavior of three groups of beagles: (1) kenneled dogs living in a restricted environment with limited human contact (*N* = 78), (2) family dogs living in human families as pets (*N* = 37), and (3) adopted dogs born in the kenneled population but raised in human families (*N* = 13). We found one factor comprising most of the test behaviors, labeled as Responsiveness. Family dogs and adopted dogs scored higher in Responsiveness than kenneled dogs. However, 23% of the kenneled dogs were comparable to family and adopted dogs based on a cluster analysis, indicating a similar (positive) reaction to novel stimuli, while 77% of the kenneled dogs were unresponsive (mostly immobile) in at least part of the test. To assess if the behavioral difference between the family and kenneled dogs could be due to genetic divergence of these two populations and/or to lower genetic diversity of the kenneled dogs, we analyzed their genetic structure using 11 microsatellite markers. We found no significant difference between the populations in their genetic diversity (i.e., heterozygosity, level of inbreeding), nor any evidence that the family and kenneled populations originated from different genetic pools. Thus, the behavior difference between the groups more likely reflects a G × E interaction, that is, the influence of specific genetic variants manifesting under specific environmental conditions (kennel life). Nevertheless, some kenneled individuals were (genetically) more resistant to social and environmental deprivation. Selecting for such animals could strongly improve the welfare of kenneled dog populations. Moreover, exploring the genetic background of their higher resilience could also help to better understand the genetics behind stress- and fear-related behaviors.

## Introduction

In the last two decades the social competence of the domestic dog has received wide attention both from theoretical and empirical perspectives [see review in (1, 2)]. While it is clear that both genetic and developmental effects play some role in the emergence and manifestation of these behaviors, we still do not fully understand the effect of the human social environment on dog behavior. Most of our current knowledge has come from comparing extensively socialized wolves and dogs (3, 4), or from testing dogs living in human homes as pets. Pet dogs nowadays are mostly regarded as a family member or friend [e.g., (5)], and living close to and interacting with humans on a daily basis constitute the norm. To understand the significance of socialization and environment-related factors on the behavior of dogs, we also need to investigate dogs with limited opportunity to interact with humans (1), i.e., feral, shelter, and kenneled dogs.

Feral or village dogs and shelter dogs, while living with limited human contact, usually have a diverse genetic and environmental background and past experiences with humans [see review in (6)]. On the other hand, dogs bred and kept solely for breeding or research purposes (“kenneled dogs”) are kept and handled under standardized conditions, and are usually of the same breed, thus the effects of environmental and breed-specific genetic variability on their individual behavioral variability is small. Most of these dogs are raised and live in intraspecific groups, and their interaction with humans is mostly limited to feeding, cleaning their enclosure, and research procedures. These factors make them particularly suitable for exploring the relative effects of environment and experience with humans on the behavior. Moreover, the standardized keeping conditions are also ideal for studies that require longitudinal design, systematic testing, or extensive training. Thus, kenneled dogs are also often used in research on cognition, such as the effect of age on different cognitive functions (7–10), or the effect of different training schedules on the acquisition of a task (11).

However, kennel keeping conditions do not represent the dogs' natural environment, which raises the question how valid these results are for the general dog population. Studies have shown that the social and spatial restrictions of the kennel environment could lead to poor welfare [e.g., (12, 13)], and different stress handling, especially when subjected to novel stimuli [e.g., (14–16)]. For example, Clark et al. (17) reported increased frequency of abnormal behaviors after 12 weeks of being subjected to standardized laboratory housing and keeping conditions. Early studies also reported that the lack of exposure to social and environmental stimuli during the early life could lead to the development of persistent behavioral abnormalities [i.e. “kennel-dog syndrome” (18–21)]. More recent studies showed that, contrary to the dogs tested in the 50's and 60's, present day kenneled (laboratory) dogs are ready to interact with humans and had no problem in quickly integrating into human families (22, 23). However, most of the dogs involved in these studies had regular affiliative and communicative interaction with their caretaker [e.g., were taken on walks, were petted and played with, participated in short training sessions, and so on (22, 23)]. It should also be noted that in these studies, dogs that were not willing to interact with the experimenter were excluded, so they may underestimate the risk of showing abnormal behaviors. In contrast, McMillan et al. (24) found that dogs adopted from commercial breeding establishments display numerous behavioral abnormalities (including extreme fears and phobias) even after 2 years of adoption, similar to the “kennel-dog syndrome.”

Taken together, while kenneled dogs could provide an opportunity to investigate the effect of limited past experience with human affiliative and communicative signals on the adult dog's behavior, their mere existence raises ethical and welfare concerns (13, 25, 26). While the wellbeing of animals used in research are increasingly recognized as a major issue of the scientific community, little is known about the general effect of kennel environment on the animal behavior, that is, how different the behavior of kenneled dogs is compared to that of pet dogs. Systematic comparison of kenneled and family dogs' behavior is still missing.

The first aim of the present study was to compare the behavior of beagle dogs living in kennels (such as in laboratory facilities), and those living in families, in a series of situations. Particular emphasis was placed on the dogs' reaction to novel stimuli and their social behavior toward humans—two fields where the kenneled dogs lack experience. Secondly, we also investigated how uniform the behavior of the kenneled dogs is. Previous studies reported large individual differences in the way laboratory animals adapt to the kennel environment [e.g., (17, 27)], however, one might also expect small individual variability among the kenneled dogs due to their uniform socialization and keeping environment and (potentially) smaller genetic variability.

Potential behavior differences between kenneled and family dogs could be due to two factors (and their interaction): (1) difference in their socialization, keeping environment, and past experiences, (2) genetic differences between the two populations. To be able to distinguish between these potential causal factors, we also tested dogs that were bred as kenneled dogs, but had been adopted by families at 8 weeks of age and raised as pet dogs. These “adopted dogs” genetically belong to the kenneled dog population, but their keeping environment and experiences are those of a family dog. If adopted dogs are more similar to kenneled dogs in their behavior, it would suggest a stronger genetic influence behind the behavior differences between family and kenneled dogs. By contrast, higher behavior similarity between adopted and family dogs would suggest a stronger influence of socialization and keeping environment.

Finally, we analyzed if there are population genetic differences between the kenneled and family dog populations. The kenneled dogs used in research (including the dogs assessed in the current study) are purpose-bred dogs and originate from a smaller breeding stock than family dogs (where inbreeding is strongly discouraged). Thus, the kenneled dog population may show an increased level of inbreeding and homozygosity, which could lead to inbreeding depression. Moreover, it is also possible that due to the limited outbreeding of the kenneled stock, the kenneled and family beagles have become genetically separated, similar to the show and working lines in some breeds (28). To investigate possible population genetic differences between the family and kenneled dogs, we analyzed (1) the distribution of allelic variations of neutral markers in the two stocks, (2) the level of inbreeding, and (3) the population structuring effects. If sufficiently large degree of population genetic substructure can be found in the examined sample pool, it highlights the possibility that the behavior differences can be traced back (at least partially) to the different genetic origin of the family and kenneled beagles.

## Materials and Methods

### Subjects

Our subjects were 78 beagle dogs from two institutes, Institute 1 and 2 (kenneled dogs, 67% males, 2.45 ± 1.28 years, bred by the same breeder), 37 privately owned beagle dogs (family dogs, 49% male, 3.28 ± 2.76 years), and 13 beagle dogs born in Institute 1 and adopted by volunteer families at the age of 8 weeks (adopted dogs, 46% males, 1.10 ± 0.09 years).

Compared to the complex environment of the dogs living in human families, kenneled dogs in our study were housed with limited contact with the outside world. They were kept in intra-specific groups of 2 to 10 dogs, had no environmental enrichment in their kennels, their interaction with humans was limited to daily feeding and cleaning, and monthly routine veterinary check. More detailed information about the demographic characteristics and keeping conditions of the three groups of dogs are summarized in the Supplementary Table 1.

Only a subset of kenneled and family dogs (*N* = 68 kenneled, *N* = 27 family dog) were used in the population genetic analyses due to missing DNA samples and because some samples were not successfully genotyped, or had missing or ambiguous genotype at some markers involved in the study.

### Phenotyping

Altogether six female experimenters carried out the behavioral tests, assigned randomly to the individuals. The test battery [FIDO Personality test, (29)] took ~30 min to complete, and comprised of 13 subtests. The order of the subtests was the same for all dogs, however, the location of the test and the involvement of the experimenters varied both among and between the dog groups.

### Differences in the Experimental Setup Between the Dog Groups

Because kenneled dogs did not have a primary caretaker, the behavior test of all kenneled dogs involved two experimenters: Experimenter 1 (unfamiliar to the dog) carried out the test while Experimenter 2 (after a short familiarization, see below) played the role of the “owner.” In order to match this setup, a subset of the family dogs (*N* = 13) were tested in a similar way, while the real owner stood close by. For the remaining 24 family dogs and in the cases of all the adopted dogs, the owner participated in the test.

Regarding the location, the kenneled dogs from the two institutes had different housing conditions, dogs from Institute 1 had permanent access to outdoor runs, while dogs from Institute 2 were kept indoors (see Supplementary Table 1). In order to match their accustomed location, kenneled dogs from Institute 1 (*N* = 55) were tested outdoors, in an unfamiliar, undisturbed area, while dogs from Institute 2 (*N* = 23) were tested indoors, in a 2.5 × 4 m testing room unfamiliar to the dogs. Again, to match this setup, the family dogs were tested in an unfamiliar outdoor area, while the adopted dogs were tested in an unfamiliar indoor testing room (5 × 6 m).

### Procedure

The test battery is presented in Mirkó et al. (29), here we describe the subtests briefly. In the cases of the kenneled dogs and the family dogs where Experimenter 2 played the role of the owner, a short (~10 min) familiarization with Experimenter 2 preceded the behavioral test itself. This episode included gently talking to and petting the dog, taking the dog for a short walk (getting used to the leash), and offering food to the dog.

#### Spontaneous Activity

The owner (the real owner or Experimenter 2, hereafter: O) stands still without paying special attention to the dog, while holding the dog on a leash (1.5–2 m). The dog is allowed to move freely within the range of the stretched leash and is not corrected or rewarded for any behavior. This test lasts for 1 min. Experimenter 1 (hereafter: E) stays at a distance of at least 3 m from the dog without paying any attention to the dog.

#### Greeting

The O stands motionless next to the dog and holds the leash. E approaches them in a friendly way, stops out of reach of the dog and waits for 3 s. If the dog is not aggressive/does not avoid E, she steps to the dog and pets the dog's head and back. Then E steps away and waits for another 3 s, then pets the dog again. If the dog avoids E (without aggressive display), E stays outside the reach of the leash, crouches and tries to call the dog to her. If the dog finally approaches E, she pets the dog, and follows the instructions above. If the dog does not respond to the calling the test is terminated after 30 s. If the dog is aggressive (growling, barking), E remains out of reach of the leash, talking continuously to the dog for 10 s and then terminates the test.

#### Pendulum Test

The O stands behind the dog holding the leash, and does not interact with the dog.

##### Phase 1

To increase the dog's motivation to obtain the sausage during the following test, E moves a small piece of sausage in front of the dog from left to right three times, then puts the piece of sausage in front of the dog. This procedure is repeated with E moving the sausage in the other direction.

##### Phase 2

E stands in front of the dog, and swings a sausage (10–12 cm long), attached to a 30 cm long string, in front of the dog's nose just out of its reach for 30 s. After that E gives the dog a piece of sausage.

#### Separation

The dog is tethered to a tree or to the wall on a long (~3 m) leash, while O goes away and hides behind an object (a big tree or an open door). After 1 min E approaches the dog and greets it (see description above: 2. Greeting). Then E initiates play with a tug for 30 s, then steps back to the camera. After 1 min, the O comes back and greets the dog (see description above). Afterwards O also initiates play with a tug for 30 s.

#### Ball Play

The dog is attached to a long (~ 3m) leash (or unleashed in the case of some family dogs). The O throws a tennis ball 3 times a few meters away and encourages the dog to grab the ball and bring it back to him/her.

#### Problem Solving

E puts a small (22 × 14 × 15 cm) plastic cage in front of the dog, giving the dog the opportunity to explore the cage. The cage has a narrow hole on the bottom (too small for the dog the reach inside by nose or paw), so when a piece of food is placed in the cage it can be retrieved by rolling or pushing over the cage. As a pre-training, E puts a piece of food next to the location of the hole, outside the cage so the dog can eat it. During the trial, the O stands 1 m behind the dog, holds the leash and is allowed to encourage the dog but only verbally. E puts a piece of food in the middle of the cage, and puts the cage in front of the dog, then steps back behind the camera. The dog then is allowed to approach the cage and has 60 s to get the food by any means. The trial ends when the dog gets the food, or after the 1 min (in which case E gives the food to the dog). The trial is repeated once with the same setup.

#### Bone Take Away

For this test we use a ham bone attached to a string. The O gives the bone to the dog and encourages it to chew it (afterwards he/she stands behind the dog holding the leash and does not interfere). If the dog starts to chew the bone, E puts on an artificial hand (a plastic tube covered with a coat sleeve, and a textile glove filled with plaster), waits for 5 s, then approaches the dog from the front, but stays out of the reach of the dog.

Steps of taking the bone away:

1. E crouches and pets the dog's head and back with the artificial hand 3 times. She does not talk to the dog.
2. E says “please” and reach toward the bone with the artificial hand.
3. E touches the bone with the artificial hand for 3 s.
4. E pulls the bone by the string, while the artificial hand is continuously on the bone.

The test is terminated if the dog (a) is not motivated to chew the bone, (b) allows E to take the bone away, or (c) shows severe aggression (i.e., snaps at the hand). If E could not take the bone away from the dog, the O is asked to do it. The test is repeated once more with the same setup.

#### Threatening Approach

O stands motionless next to the dog and holds the leash. E, standing 4–5 m from the dog, starts approaching the dog slowly, with a slightly bent upper body and looking steadily into the eyes of the dog without any verbal communication. If the dog looks away from E, she tries to attract the dog's attention by making some noise (cough, stamping). The test is terminated if (a) E reaches the dog, (b) the dog approaches E in submissive or friendly manner, (c) the dog barks/growls at the E continuously, (d) the dog hides behind the O. After the approach is terminated E steps back to her starting position, crouches, and calls the dog in a friendly way.

#### Umbrella

O stands motionless next to the dog and holds the leash. E approaches the dog from the front with a closed umbrella in her hand (with the end of the umbrella pointed toward the dog). When she is within ~1 m of the dog, she opens the umbrella, lifts it up slowly above her head, then puts the umbrella on the ground, and steps away. Then the O is asked to walk the dog next to the umbrella. If the dog avoids the umbrella, O steps next to the umbrella, touches it and calls the dog.

#### Lying to the Side

The O commands the dog to lie down or gently puts the dog in a lying position. Then O crouches down next to the dog and turns the dog onto its side. If the dog refuses to lie on the side, the test is terminated after 60 s. Otherwise, O tries to keep the dog in this position for 30 s, petting and talking to the dog is allowed. If the dog gets up before the 30 s, the test restarts. If the dog manages to get up the second time, the test is terminated.

#### Food Choice

O stands still and holds the dog on the leash.

##### Phase 1

E turns her back to the dog and puts 1 piece of food on a white plastic plate, and eight pieces on another plate. E shows both plates to the dog then steps back 2 m and places both plates on the ground simultaneously (1.5 m apart from each other). Then E steps back, and tells the O to let the dog free to make a choice.

##### Phase 2

Same as phase 1, but before stepping back from the plates E crouches down next to the plate with one piece of food, picks up the food, imitates eating and says “Hmm-mmm,” then puts the food back on the plate.

#### Hiding

E holds the dog on the leash, meanwhile the O walks away out of the dog's sight (hides behind a large tree 15–20 m away from the dog or goes out of the testing room). After 30 s, independently from the orientation of the dog, the E relaxes the leash or removes the leash and says “Go!”. If the dog does not start to move within 5 s, the E encourages it gently by nudging the rear end of the dog. If the dog still does not start to move or moves in a different direction than O's hiding place, E asks the O to call the dog. If the dog still does not approach the O, the O comes back, while continuously calling the dog.

#### Spontaneous Activity 2

The procedure was the same as the first subtest.

### Behavioral Variables

All tests were video-taped, and 50 behavioral variables were scored on a 0–3 scale in each subtest (for detailed definitions see Supplementary Table 2). To assess the inter-observer reliability of the scoring *N* = 49 videos (38%) were coded by two observers.

### DNA Sample Collection, DNA Isolation and Storage

Buccal samples were collected from dogs in a non-invasive way by rubbing a pair of cotton swabs to the inner side of the dog's mouth (30). DNA was extracted from buccal swabs by ethanol precipitation technique (31). The concentration of DNA solutions was determined using a NanoDrop 2000 spectrophotometer. Extracted DNA samples were stored at −20°C long term after quantitation.

### Genotyping of Population Samples

For analyzing the genetic structure we used ten autosome and one X chromosome localized microsatellite markers, also called STRs (Short Tandem Repeats). The analyzed STR markers are 90–350 bp long non-transcriptional variable number of tandem repeat sequences at certain points of the genome. In the human genome, thousands of such microsatellite markers have been identified (32), accounting for nearly 3% of our DNA (33) and the same ratio is expected in the dog genome (34). Microsatellites are commonly used in population genetic studies in canine species (35, 36), in forensic caseworks (37, 38) and in conservation biology (39, 40) due to their Mendelian inheritance and informativeness. They are neutral, multi-allelic and have greater discriminatory power than biallelic markers such as SNPs. Their dispersion is consistent in canine populations and the average mutation frequency (that is around 10^−3^) is much more suitable to examine recent changes in the population structure compared to SNPs. Their outstanding usability is well illustrated by the fact that there are several commercially available kits for laboratories for canine DNA testing (i.e., Canine ISAG STR Parentage Kit), breeders use them for parentage testing (37, 41), and they are part of the everyday forensic examinations (38, 42). The allelic nomenclature applied for the 11 canine microsatellite markers is based on the recommendations of the International Society of Forensic Genetics (ISFG) and of the European DNA Profiling Group (EDNAP) concerning the nomenclature of human STRs (43, 44).

The chromosome localization, repeat structure and PCR primer sequences of the analyzed markers are summarized in Supplementary Table 3. The investigated autosome STRs are located on distinct chromosomes to assure their independency for the statistical analyses. The examined markers were PCR co-amplified in two separated multiplex reactions—so-called MiniPlex I-II—and the primers used were the same as published by Zenke (45). For the precise allelic determination and genotyping we have optimized the MiniPlex I-II PCR systems to our laboratory conditions. Primer concentrations were changed and optimized to achieve efficient amplification and fidelity. DNA template concentration was tested to determine the minimum quantity of DNA needed for successful multiplex amplification. Optimized conditions for the two 6-Plex PCR systems were established in 20 μl reaction volume as follows: 1x concentration of PCR Buffer II, 2.5 mM MgCl_2_, 1.0 mM dNTP of each, 2U of AmpliTaq Gold^TM^ DNA polymerase (Life Technologies), 1 ng BSA (Bovine Serum Albumin), 1–5 ng DNA template and appropriate primer concentrations. Primer concentrations and PCR amplified DNA fragment lengths are shown in Supplementary Table 3. The two multiplex PCR were performed by the same cycling conditions in a GeneAmp PCR 9700 thermocycler (Applied Biosystems) as follows: 95°C for 11 min, 30 cycles of 45 s at 94°C, 45 s at 58°C and 45 s at 72°C, with final extension at 72°C for 45 min. The 58°C annealing temperature was optimal in case of both multiplexes. PCR products were monitored by agarose gel-electrophoresis in a GNA-100 submarine electrophoresis tank with an EPS 3500 XL power supply (Pharmacia Biotech) using 2% agarose gels (Agarose, MetaPhor^TM^). Visualization of amplified DNA fragments was carried out with GelRed® staining (Biotium) followed by UV detection. The amplified PCR products of both 6-plex systems were separated and detected on an automatic 4-channel capillary-electrophoresis system of ABI 3130 Genetic Analyzer (Applied Biosystems). For capillary-electrophoresis 3130-POP4 polymer, 36 cm capillary array and default instrument settings were applied, and for fragment sizing we used GeneScan500-LIZ® internal size standard (Applied Biosystems). The fragment lengths and allelic designation were determined using GeneMapper® ID v3.2.1 software (Applied Biosystems), based on the number of the repeat motifs. For accurate allelic designation we created in-house BinSets in the GeneMapper software involving each analyzed marker.

### Statistical Analysis

#### Behavioral Analyses

The 50 behavioral variables were subjected to a two-step data-reduction method [similar to (46)]. Two steps were needed because of the relatively high number of variables and the high correlations between the variables within subtests. In the first step Principal Component Analyses (with Varimax rotation) were run for each subtest separately. The two Spontaneous activity tests were analyzed together since only one variable was measured in each. The resulting components were then subjected to an exploratory factor analysis (EFA) with Varimax rotation. The number of factors retained was decided by running a Parallel analysis, using the syntax program for SPSS provided by (47), and subtest-components that failed to load with at least 0.32 on any EFA factor were excluded in a stepwise manner (48). Cronbach's α was calculated to assess the internal consistency of the resulting factors. Inter-observer reliability was assessed using intraclass correlation (ICC, 1,k absolute agreement). While the residuals of the obtained factor scores were close to be normally distributed the assumption of homogeneity of variance across the dog groups was strongly violated, so we used non-parametric tests for further analyses.

First, we ran four preliminary analyses. We tested the possible differences between the kenneled dogs from the two Institutes (as their keeping and testing environment were different), and between the two sub-groups of family dogs (tested with the owner vs. tested with the experimenter) using Mann-Whitney U tests. Moreover, we also investigated the effects of sex and age on the behavior factor scores separately for the family, adopted, and kenneled dog groups. First because the dog groups were not matched for these two factors, so they could be a source of the behavior difference between the groups, and second, to investigate if sex or age could be related to the behavior variability of kenneled dogs. Differences between males and females were investigated using Mann-Whitney U tests, the effect of age was analyzed using Spearman correlations (however, adopted dogs were not investigated in the age-association since all dogs were ~1 year old at the time of testing).

Second, to test the behavioral differences between the kenneled, adopted and family dog groups we used Kruskal-Wallis test. Effect sizes for the behavioral comparisons were estimated with eta squared (η^2^). To investigate how uniformly the dogs behaved within each dog group (family, adopted, kenneled dogs) we compared the behavioral variance between the dog groups using Levene's test for equality of variances. Furthermore, we also performed two cluster analyses. The first one only on the kenneled dogs, to investigate the behavioral variability among them, the second one on all dogs to assess whether the family, adopted and kenneled dogs form distinct groups based on their behavior. For the clustering we used Ward's method. It is a hierarchical agglomerative method that starts with each case as a cluster on its own, then merges pairs of clusters step-by-step, starting with the lowest merging cost (that is, how much the sum of squares will increase upon merging), until all cases are in one cluster (49). The number of clusters retained (K) were decided based on how much the merging cost changes with each new cluster formed (relative to the previous cost), decreasing K until the cost suddenly jumps up (50). SPSS 22.0 for Windows was used for the statistical analyses.

#### Population Genetic Analyses

From the genotype data exported from GeneMapper, we determined the allele and genotype counts and frequencies, and PIC values [Polymorphism Information Content, (51)] using an Excel macro. Calculation of the expected and observed heterozygosity values at each locus (H_Exp_, H_Obs_) were made using the Arlequin 3.5 software (52). Arlequin was also used for testing the deviation from the expectation of the Hardy-Weinberg equilibrium (*HWE*) by a modified Fisher's exact test. Bonferroni correction was used account for the effect of multiple tests on genetic equilibrium, setting the threshold of the significance level to *p* = 0.0045 (53). To determine the intra- and inter-population genetic variations (i.e., population genetic structure) Wright's F-statistics (54) and molecular variance analysis (AMOVA) were calculated in Arlequin 3.5 software (55, 56). Inbreeding coefficient (F_IS_) and population substructure parameter (F_ST_) were calculated according to Weir and Cockerham (57). We also used the Admixture model in the Structure 2.3.4 software (58, 59) to investigate the possible population structure in our sample at the individual level, based on all the markers. This analysis identifies genetically distinct subpopulations on the basis of allele frequency patterns. We ran the model for 5,000 iterations after a burn-in of 50,000 iterations, and pre-defined the number of subpopulations (K) as 2, because we aimed to check if family and kenneled dogs split up into different genetic subgroups. The model was run in four ways, taking into account none, only one, or both of the following factors, as prior information to assist the clustering: the population subdivision (PopData; family or kenneled dog), and the location of the sampling (LocPrior, Institute 1, Institute 2, family dog).

## Results

### Exploratory Factor Analysis

The raw data of the behavioral variables and factors are given in Supplementary Table 2. The subtest-level PCAs resulted in 17 subtest-level components which were subjected to the EFA, and the parallel analysis suggested two factors to be extracted. Three subtest-level components did not load on any of the factors with > 0.32 and so were excluded. According to the factorial matrix (Table 1) all the remaining 14 subtest-level components loaded on the first factor, and only two of them cross-loaded on the second factor. This suggests that there might be only one background factor accounting for most of the variance we measured in the test, therefore we retained only the first factor for further analyses. Given its constitution of a broad variety of social and non-social behaviors we labeled it as Responsiveness. A high score in this factor corresponds to a more positive reaction to various stimuli (i.e., faster approach, higher interest, more attention), while a low score could mean both indifference and more negative (i.e., active or passive fear) reaction. The internal consistency and inter-observer reliability of Responsiveness were both high (Cronbach's α = 0.930; ICC = 0.906, F_48, 49_ = 10.685, *p* < 0.001).

**Table 1:** Results of the exploratory factor analysis.

**Raw variables**	**Variable loading**	**Subtest component**	**Factor 1**	**Factor 2**
Latency of approaching E	0.627	Separation C1	**0.900**	0.094
Latency of following E	0.591			
Duration of playing with the E	0.813			
Latency of approaching O	0.666			
Latency of following O	0.674			
Duration of playing with the O	0.729			
Duration of moving	0.649	Pendulum test	**0.865**	0.066
Duration of orientation to object	0.864			
Latency of eating	0.891			
Latency of choosing a plate1	0.973	Food choice	**0.858**	0.097
Latency of choosing a plate2	0.973			
Duration of moving1	0.862	Separation C3	**0.830**	−0.054
Duration of moving2	0.774			
Duration of orientation to cage1	0.962	Problem solving C1	**0.792**	−0.078
Duration of orientation to cage2	0.953			
Latency of success1	0.872			
Latency of success2	0.909			
Latency of approaching E	0.932	Greeting	**0.783**	−0.112
Latency of following E	0.932			
Latency of grabbing the bone1	0.908	Bone take away C1	**0.725**	−0.216
Latency of grabbing the bone2	0.924			
Releasing the bone1	0.921			
Releasing the bone2	0.910			
Final reaction	0.865	Threatening approach C1	**0.677**	−0.292
Latency of approaching E	0.859			
Reaction to umbrella	−0.840	Umbrella	**0.673**	−0.270
Latency of approaching umbrella	0.840			
Duration of orientation to O	0.715	Hiding	**0.668**	**0.459**
Duration of vocalization	0.742			
Latency of approaching O	0.789			
Speed of approach O	0.764			
Intensity of playing	0.919	Ball play	**0.625**	0.239
Number of following the ball	0.834			
Number of retrieving the ball	0.900			
Number of giving out the ball	0.805			
Duration of orientation to O1	0.888	Separation C2	**0.524**	**0.421**
Duration of orientation to O2	0.915			
Duration of moving1	0.832	Spontaneous activity	**0.514**	−0.173
Duration of moving2	0.832			
Aggression	0.945	Threatening approach C2	**0.425**	0.244
Duration of vocalization	0.935			
		Eigenvalue	7.572	1.286
		Explained variance	54.10%	9.20%
		Cronbach's α	0.930	0.730

*The Eigenvalues, explained variance and Cronbach's α values are presented at the end of the table. C1, C2, etc. after a subtest's name indicates that more than one component was derived from that subtest. The raw variables which made up each subtest-level component are also presented to ease the interpretation of the factors. E, experimenter; O, owner; Loadings > 0.32 are in boldface. Only Factor 1 was retained for further analyses*.

### Comparing Subgroups in Responsiveness

No significant difference was found between the kenneled dogs tested in the two institutes (*N* = 55, *N* = 23, *z* = 0.674, *p* = 0.500, η^2^ = 0.006), nor between the family dogs tested with the owner vs. with the experimenter (*N* = 24, *N* = 13, *z* = 0.827, *p* = 0.408, η^2^ = 0.018). Therefore, we analyzed the kenneled dogs from the two institutes as one group, as well as merged the two sub-groups of family dogs.

Regarding sex differences, no difference was found in the case of family and adopted dogs (*p* > 0.5 for both), and only a weak effect was found in the kenneled dogs (*N* = 78, *z* = 1.966, *p* = 0.049, η^2^ = 0.05) with females being more responsive than males.

**Figure 1:**
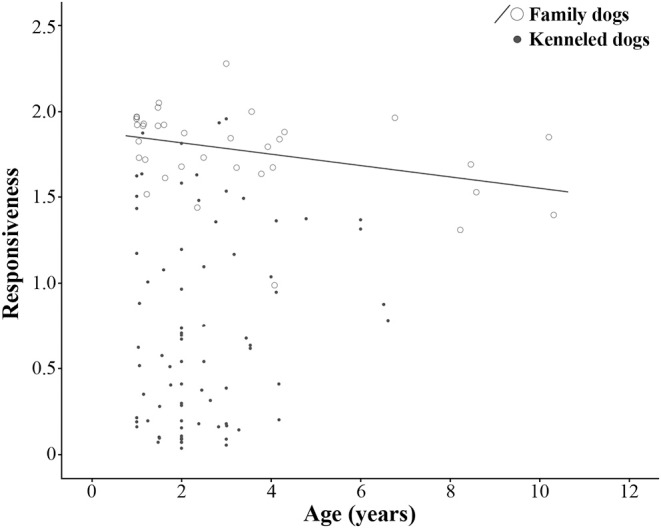
Association between age and Responsiveness factor in family and kenneled dogs. Adopted dogs were not included in the age-association analysis due to their narrow age range (0.93–1.30 years).

No age effect was found in the case of kenneled dogs (*N* = 78, ρ = 0.106, *p* = 0.354). However, we found a moderate negative correlation between age and Responsiveness in the case of family dogs (*N* = 36, ρ = −0.433, *p* = 0.009), older dogs were less responsive than younger ones (Figure 1).

### Behavior Differences Between Dog Groups

**Figure 2:**
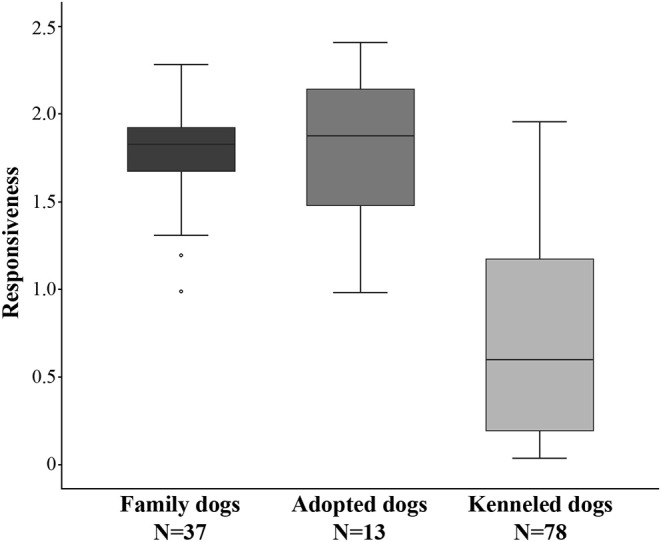
Differences between family, adopted, and kenneled dogs in Responsiveness. The family and adopted dogs differed from kenneled dogs (*p* < 0.001 for both).

We found a strong difference in Responsiveness between the family, adopted, and kenneled dogs (*N* = 128, χ^2^ = 67.767, *p* < 0.001, η^2^ = 0.528). *Post-hoc* tests indicated no difference between family dogs (*N* = 37, median = 1.83) and adopted dogs (*N* = 13, median = 1.88) (*p* = 1.000, η^2^ = 0.002), however, both were more responsive than kenneled dogs (*N* = 78, median = 0.60) (*p* < 0.001, η^2^ = 0.499; *p* < 0.001, η^2^ = 0.252, respectively) (Figure 2).

The three groups also differed in their variance (F_2, 125_ = 16.924, *p* < 0.001). Family dogs had smaller variance (VAR = 0.066) than adopted (VAR = 0.194) and kenneled dogs (VAR = 0.324) (F_1, 48_ = 9.656, *p* = 0.003; F_1, 113_ = 32.689, *p* < 0.001), while no significant difference was found between the latter two groups (F_1, 89_ = 1.914, *p* = 0.170).

### Cluster Analysis

#### Kenneled Dogs

To assess the behavior variability among the kenneled dogs we also performed hierarchical cluster analysis on the basis of the Responsiveness factor. The merging cost showed a steady increase from *K* = 77 to *K* = 3, each cost being 1.1–1.7 times higher than the previous one. The largest increase was observed at *K* = 2 where the merging cost was >3 times higher than at *K* = 3, which indicates that there are three markedly different clusters among the kenneled dogs.

The least responsive cluster (Cluster 1) contained *N* = 34 dogs (43.6%). These dogs remained largely unresponsive throughout the whole test, spending much time immobile, occasionally showing overt avoidance. Cluster 2 (*N* = 26, 33.3%) also reacted with immobility at the beginning of the test, but at one point they started moving and interacting with humans and the environment. The most responsive cluster (Cluster 3) contained *N* = 18 dogs (23.1% of the kenneled dogs), these dogs reacted largely positively to the environmental and social stimuli from the beginning on.

#### All Dogs

**Figure 3:**
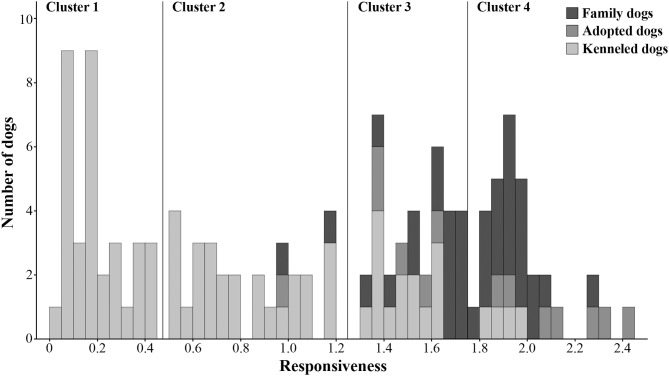
Histogram of Responsiveness. The four clusters identified by hierarchical cluster analysis are separated by horizontal lines. The three dog groups (family, adopted, kenneled) are shown in different colors.

**Figure 4:**
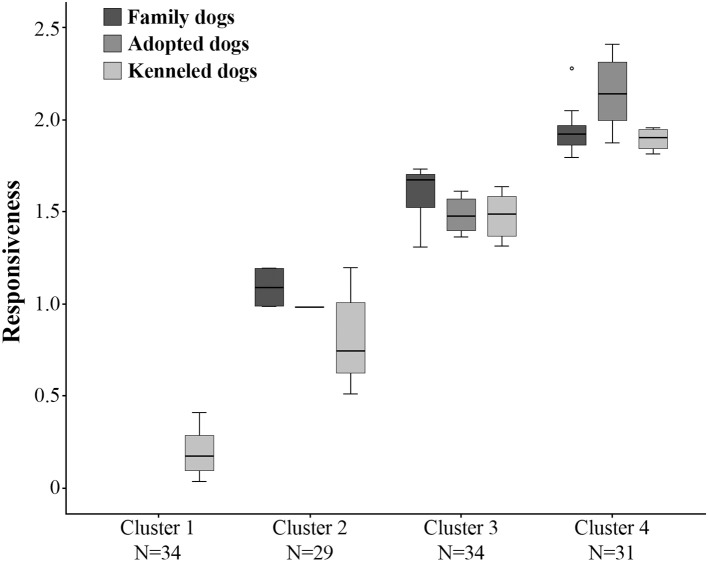
Differences between the four clusters in Responsiveness. All clusters differ from each other at the level of *p* < 0.001. The three dog groups (family, adopted, kenneled) are shown separately within each cluster.

To investigate whether the family, adopted and kenneled dogs form distinct groups based on their behavior, we repeated the cluster analysis, this time including all dogs. This analysis resulted in four clusters (see Figure 3, Supplementary Figure 1). Cluster 1 was the same as in the previous analysis, containing only kenneled dogs with markedly low responsiveness (labeled as “unresponsive”). Cluster 2 (labeled as “moderately responsive”) also contained the same kenneled dogs as in the previous analysis, but additionally one adopted and two family dogs were also clustered here. The responsive dogs, however, were divided into two clusters, and 94% of the family and adopted dogs were grouped in these two clusters. Cluster 3 (“responsive”) contained 15 family, five adopted and 14 kenneled dogs, while Cluster 4 (“highly responsive”) included 20 family, seven adopted and four kenneled dogs. The clusters differed strongly in Responsiveness (Kruskal-Wallis test, *N* = 128, χ^2^ = 118.974, *p* < 0.001, η^2^ = 0.935; all pairwise comparisons: *p* < 0.001, η^2^ = 0.734–0.737, Figure 4). The characteristics of the kenneled, adopted and family dogs in the different clusters can be found in the Supplementary Table 4; we found no significant association between the dogs' cluster membership and sex, age, or subgroup.

### Genetic Diversity in the Family and Kenneled Dogs

Individual genotype data, and allele and genotype frequencies are given in Supplementary Table 2. The accurate genotypes of all detected alleles at each locus have been clustered according to their lengths. There were no significant differences in the fragment sizes within each assigned category using a ± 0.5 bp window. Due to the high resolution power of the applied capillary electrophoresis system the intermediate sized alleles could have been separated unambiguously (i.e., WILMS-TF and FH2584). Only a minor part of the samples had to be re-analyzed mainly because of the weak signals or drop-outs of some alleles. As the replicates were all concordant we accepted them as true genotypes. The received DNA profile of each specimen was identified unambiguously and none of the 95 animals were identical in genotype at the 11 loci (see Supplementary Table 2).

In the case of allele frequencies we did not find any significant differences between the family and kenneled beagles (Supplementary Table 2). Some rare alleles at almost each locus have been observed only in one of the stocks, probably due to the small sample size. Surprisingly, we detected only two alleles on vWF.X locus in both stocks, even though in the case of other dog breeds this locus was proved to be more polymorphic, harboring multiple alleles. This suggests that vWF.X has low informative value for population genetic studies in the beagle breed (in Hungary). As for the genetic equilibrium, nearly all loci in both stocks and in the whole population agreed with the expectation of the Hardy-Weinberg Equilibrium (*HWE*) (Table 2). This was rather unexpected because of the small sample size and supposed inbreeding of the kenneled beagle stock. The only deviation from the equilibrium was found in the X chromosome-linked FH2584 marker in the kenneled stock and in overall, and it can be due to the rather few females in the kenneled sample, that causes a reasonable variance in the observed allele counts and in allele frequencies at this locus. Based on the obtained allele frequencies we calculated the expected and observed heterozygosity (H_Exp_, H_Obs_), as well as the polymorphic information content (PIC) values for each stock (Table 2). PIC is lower than H if the sample consists of non-related individuals (51). The lowest PIC and heterozygosity values were measured at the locus vWF.X which can be explained by the fact that only two alleles were detected at this locus.

**Table 2:** Expected and observed heterozygosities, PIC and Hardy-Weinberg Equilibrium *p*-values.

**Marker**	**Stock**	**H_**Exp**_**	**H_**Obs**_**	**HWE (*P*-value)**	**PIC**
PEZ1	KEN	0.678	0.618	0.356	0.611
	FAM	0.646	0.741	0.027	0.566
	ALL	0.690	0.653	0.096	0.626
PEZ5	KEN	0.690	0.676	0.810	0.629
	FAM	0.616	0.593	0.304	0.520
	ALL	0.673	0.653	0.488	0.609
PEZ3	KEN	0.772	0.691	0.038	0.733
	FAM	0.760	0.630	0.795	0.711
	ALL	0.768	0.674	0.034	0.731
PEZ21	KEN	0.568	0.456	0.118	0.500
	FAM	0.643	0.593	0.297	0.582
	ALL	0.589	0.495	0.092	0.527
PEZ16	KEN	0.788	0.779	0.207	0.750
	FAM	0.811	0.926	0.571	0.771
	ALL	0.805	0.821	0.644	0.774
REN124F09	KEN	0.520	0.500	0.186	0.475
	FAM	0.744	0.926	0.573	0.682
	ALL	0.606	0.621	0.865	0.559
PEZ19	KEN	0.595	0.618	0.922	0.511
	FAM	0.402	0.370	0.541	0.360
	ALL	0.561	0.547	0.687	0.488
WILMS-TF	KEN	0.889	0.853	0.072	0.871
	FAM	0.805	0.778	0.579	0.764
	ALL	0.873	0.832	0.212	0.856
FH2054	KEN	0.747	0.676	0.233	0.706
	FAM	0.611	0.519	0.646	0.552
	ALL	0.726	0.632	0.228	0.689
FH2584	KEN	0.797	0.926	0.005	0.806
	FAM	0.792	0.704	0.151	0.674
	ALL	0.808	0.863	0.001	0.778
vWF.X	KEN	0.198	0.221	1.000	0.177
	FAM	0.307	0.296	1.000	0.256
	ALL	0.230	0.242	1.000	0.202

*N = 68 for kenneled (KEN), N = 27 for family (FAM), and N = 95 for overall (ALL). p < 0.0045 (Bonferroni-corrected α)*.

To test possible inbreeding in the two stocks we calculated F_IS_ values (often referred as inbreeding coefficient) at each marker (Table 3). Surprisingly, neither heterozygosities nor F_IS_ values differed remarkably from an outbred population. Only at the PEZ21 locus in the kenneled stock was the F_IS_ value significant (when determined using conventional F-statistics), but this was not supported by molecular variance analysis. When analyzing all markers together, the overall inbreeding coefficient values were not significant in either stock, which means no significant effect of inbreeding was found in any of the stocks, nor in the whole (analyzed) beagle population.

**Table 3:** Conventional F-statistic and AMOVA values calculated in the Beagle population.

**Markers**	**Conventional F-statistic**				**Analysis of molecular variance**			
	**F_**ST**_**	**F_**IS**_ (KEN)**	**F_**IS**_ (FAM)**	**F_**IT**_**	**R_**ST**_**	**F_**IS**_ (KEN)**	**F_**IS**_ (FAM)**	**F_**IT**_**
PEZ1	0.072**	0.089	−0.150	0.095	0.013	−0.120	0.114	−0.033
PEZ5	0.014	0.020	0.038	0.038	0.001	−0.099	0.299	−0.005
PEZ3	−0.003	0.105	0.174	0.122*	−0.014	0.039	0.114	0.049
PEZ21	−0.006	0.199*	0.080	0.156*	−0.009	0.197	−0.125	0.086
PEZ16	0.033*	0.010	−0.145	−0.000	0.004	0.200	−0.318	0.039
REN124F09	0.087**	0.039	−0.251	0.027	−0.011	−0.016	−0.227	−0.121
PEZ19	0.105**	0.004	0.080	0.126	0.114**	0.013	0.017	0.127
WILMS-TF	0.021*	0.041	0.034	0.060	0.022	−0.027	0.019	0.004
FH2054	0.054**	0.095	0.154	0.158*	0.032	0.188	0.193	0.216*
FH2584	0.036**	−0.163	0.113	−0.047	0.065**	−0.567	−0.238	−0.398
vWF.X	0.012	−0.117	0.037	−0.047	0.012	−0.117	0.037	−0.047
**Overall**	0.001	0.000	−0.001	0.000	0.008	0.059	0.007	0.053

F_ST_, fixation index; R_ST_, molecular analog of F_ST_; F_IS_, inbreeding coefficient in the subpopulations; F_IT_, inbreeding coefficient in total; F_ST_, R_ST_, F_IS_, and F_IT_ values were determined at each marker in both stocks and overall. KEN, Kenneled stock (N = 68); FAM, Family stock (N = 27). Significant values are marked in bold. *p < 0.05 and **p < 0.001.

### Genetic Differences Between the Subpopulations

The F_ST_ (fixation index) values, calculated by conventional F-statistics, is a measure of genetic differentiation. It describes how much of the total genetic variance in the population can be attributed to the variance between the stocks (54, 57). The R_ST_ (often called Φ_ST_), obtained by AMOVA, is the molecular analog of F_ST_, developed specifically for STRs (55, 56). Higher F_ST_ and R_ST_ values indicate greater inter-population variance.

When we compared the family and kenneled dogs by traditional F-statistics, F_ST_ values indicated significant differences on seven out of the eleven analyzed STR loci (see Table 3). However, only two of them was confirmed by the R_ST_ (PEZ19 and FH2584). The observed small discrepancy between the two stocks can be due to possible genetic difference between family and kenneled dogs, or due to the few genotypes that were included in the analyses. We think the latter is more plausible, because the FH2584 locus is linked to the X chromosome, therefore harbors a reduced number of genotypes in males. When analyzing all markers together, neither conventional F-statistics nor AMOVA indicated significant difference between the two stocks. To further analyze the potential substructuring in the population we analyzed the multilocus genotype data at the individual level and clustered the individual genotypes into two clusters using the *Structure* software. When the location of sampling (Institute 1, Institute 2, family dog) was not taken into account, independent if we added the population subdivision (family or kenneled dog) in the model or not, the probability of the individual to be clustered in the first or second cluster was 45–55% for both kenneled and family dogs (Table 4). When only the location of sampling was considered, the family dog samples were assorted into one cluster with > ~96% probability, while the kenneled dogs were assigned into the two clusters with equal probability (49 and 51%). When both the location of sampling and the population subdivision were considered, the model indicated that the location is no longer informative (mean *r* = 1.1901), probably because of the high overlap between the population subdivision and location of sampling factors. Nevertheless, this model version classified the family dogs into one cluster with high (~92%) probability, while the kenneled dogs were assigned into the other with 66% probability.

**Table 4:** Mean estimated membership of each pre-defined population in each of the two clusters.

**Pre-defined population (*N* of dogs)**	**No PopData, no LocPrior**		**With PopData, no LocPrior**		**No PopData, with LocPrior**		**With PopData, with LocPrior**	
	**Cl 1**	**Cl 2**	**Cl 1**	**Cl 1**	**Cl 2**	**Cl 2**	**Cl 2**	**Cl 2**
Kenneled (68)	0.508	0.492	0.503	0.497	0.487	0.513	0.660	0.340
Family (27)	0.477	0.523	0.458	0.542	0.963	0.037	0.081	0.919

*With/No PopData, population subdivision (kenneled or family dog) was or was not considered; With/No LocPrior, location of sampling (Institute 1, Institute 2, family dog) was or was not considered in the model; Cl, cluster*.

## Discussion

In the present study we compared the behavior of beagle dogs living in kennels and living in families in their reaction to different social and non-social situations. Our aim was to investigate if there is any behavioral difference between family and kenneled dogs, and if so, whether this difference can be attributed to genetic difference caused by population substructuring in the kenneled and family stocks.

Although we initially expected the non-social and human-directed social behaviors to segregate into different factors, we found only one background factor accounting for most of the behavioral variance between the individuals. It suggests that in our current sample most of the variability between the individuals is related to whether or not the dog participated in the situations actively. We labeled this single factor “Responsiveness” because it describes if and how far the individual was inclined to (positively) respond to any types of stimuli, including humans, objects, or the environment. Dogs with a low score were not interested in or avoided the experimenter, did not play, did not try to obtain the food reward or bone, and, in general, had a little inclination to participate in or react to the situation/stimuli presented. Considering its constitution of a broad variety of behaviors, our Responsiveness factor could be similar to the higher-order personality traits identified in previous studies which also comprised different types of behaviors. For example, the “Activity–success” factor in (21) included activity, confidence and performance in several test situations, the “Mental stability” factor in (60) was associated with courage, nerve stability and hardness (lack of a lasting effect of a pleasant or frightening experience) across several test situations, and the “Boldness” factor (61) was composed of Sociability, Playfulness Chase-proneness and Curiosity/fearlessness.

We found a strong difference between the three dog groups in Responsiveness: family and adopted dogs both were more responsive to social and environmental stimuli than kenneled dogs. This behavior difference could not be due to unbalanced age and sex-ratio between the groups. We found a negative relationship between Responsiveness and age only in the case of family dogs, probably due to the larger age range in this group (including dogs above 8 years of age), and only a weak sex difference was found in kenneled dogs (probably due to having twice as many male than female dogs in the kenneled dog sample).

On the other hand, the high Responsiveness of adopted dogs (which were from the same genetic pool as the kenneled dogs), suggests that the kenneled dogs' lower responsiveness is more due to the effect of their different socialization, life experiences, and restricted environment, and less due to genetic influences. The importance of early socialization and rearing environment in shaping the dogs' behavior has long been documented [e.g., (62, 63)]. For example, both (21) and (64) reported that social deprivation, especially at an early age, severely disrupts normal behavior development and could lead to abnormal social behaviors, strong fear responses, and difficulties to adapt to new situations. Similarly, several earlier reports have shown that the social and spatial restrictions associated with kenneled life represent strong stressors to the dogs [e.g., (16, 65)] and could lead to a higher prevalence of undesirable (mostly fear-related) behaviors [e.g., (66, 67)].

The behavior divergence between dogs kept in families and kept in kennels was partly supported by the cluster analysis. According to that, the majority (94%) of pet (family and adopted) dogs were clustered in the “responsive” and “highly responsive” clusters (Cluster 3 and 4). These dogs behaved in a relaxed manner during the test and reacted generally positively to the different stimuli, although there was an individual variability among the pet dogs' in responsivity. By contrast, the majority (77%) of the kenneled dogs were clustered in the “unresponsive” and “moderately responsive” clusters (Cluster 1 and 2). These dogs spent much time immobile, and/or showed overt fear in at least part of the test. This confirms that the restricted (social) environment indeed has a negative effect of the kenneled dogs' responsiveness.

However, this negative effect was not universal among the kenneled dogs. Contrary to what could be expected based on their uniform keeping conditions and similar past experiences, we found a high behavioral variance among the kenneled dogs, in harmony with (17). This variability is apparent from the fact that 23% of the kenneled dogs clustered together with the pet dogs, suggesting that their behavior was not markedly different from that of dogs living in families. We hypothesize that this variability in Responsiveness among the kenneled dogs reflect a more general difference in their stress-handling. Behaviors similar to those we observed in the unresponsive dogs (i.e., excessive timidity, reduced activity and immobility) has been described in socially deprived dogs in an open field test (21), which the authors explained as a reaction to the stress caused by the overwhelming amount of new stimuli. Moreover, similar behavioral divergence has been found between two strains of pointer dogs developed through selective mating for or against showing “nervous” behaviors (68, 69). When exposed to humans or novel stimuli, dogs of the “normal” strain were friendly, active, and interested in the environment, while dogs of the ‘nervous' strain exhibited marked fear response, that is, avoidance and catatonic freezing (68–71). However, in the home kennel of the dogs the behavior of the two strains could not be distinguished from each other (72), which confirms that the fear behaviors of the nervous strain were a reaction to the stimuli, and not a permanent characteristics of these dogs. These behavioral reactions were parallel to those of the “family dog-like” and unresponsive beagles in our study. However, maybe because our kenneled beagles were not specifically selected for or against any behaviors, we also had a group of dogs with an intermediate strategy. These “moderately responsive” dogs were able to overcome their fear at one point of the test, and started moving and interacting with humans and the environment (unlike unresponsive dogs which remained unresponsive throughout the whole test).

This difference in the stress-handling could originate from two sources: (1) how readily novel social and environmental stimuli provoke stress responses in different individuals, and (2) how the different individuals cope with this stress. Regarding the former, it is possible that the “family dog-like” kenneled dogs has a generally lower sensitivity to novel (potentially stress-provoking) stimuli, enabling them to behave in a relaxed manner and react generally positively to the different stimuli presented in our study. Contrary to that, higher sensitivity in the case of the unresponsive kenneled dogs could lead to the novel stimuli being perceived as threats, triggering defense strategies to cope with them (e.g., freezing and/or escaping). Alternatively, it is also possible that the main difference between the two extremes of the kenneled dogs lies not in their general sensitivity, but in the defense strategy they use to cope with this stress, specifically, whether or not they were able to use humans as a source of security. The general defense strategy of family dogs in stressful situations is to use their owners as a safe haven (73). For kenneled dogs lacking a primary attachment figure, this strategy is normally not available, which could be one of the reasons for the observed behavioral difference between the pet and kenneled dogs. However, Gácsi et al. (74) found that in shelter dogs deprived of social contact with humans, even a short duration of human handling could lead to the formation of an attachment-like bond. Thus, it is possible that some of the kenneled dogs in our study were able to form a weak bond with the “owner” during their short familiarization, and these dogs were then able to use the “owner” as a secure base, buffering against the stress caused by the novel environmental stimuli. Contrary to that, for the unresponsive dogs, the short familiarization with their “owner” may not have been enough for establishing a bond, so for them the close presence of an (unknown) human remained another source of threat they need to cope with.

Nevertheless, the fact that both behaviorally responsive and unresponsive kenneled dogs lived in the same environment and had the same previous experiences with the presented stimuli indicates the effect of genetic factors in the regulation of this behavior. In previous studies, heritability estimates also indicated a significant genetic contribution to the variation found behavior factors similar to our Responsiveness. For example, Mental stability was found to be heritable both in German shepherds (0.25) and Labrador retrievers (0.29) (75), the heritability estimates of boldness trait was 0.25 and 0.27 for German shepherd and Rottweiler, respectively (76), while on laboratory beagles significant heritability (0.23) was found for the Social interactions factor (including variables related to proximity to and interacting with humans in an unsolvable problem paradigm) (77). Furthermore, Murphree et al. (78) showed that the behavior of crosses of the “normal” and “nervous” pointer strains was very similar to the “nervous” strain, suggesting dominant inheritance of the nervous behaviors.

Since our kenneled dog population originated from a limited breeding stock, it was possible that the behavior difference between the kenneled and family dogs reflect a population genetic divergence of the stocks. To test this we analyzed the genetic diversity and the genetic structure in the examined beagle stocks. A subset of the investigated 11 microsatellite markers have been previously examined in a canine population genetic study (36), that aimed to develop so-called “*mini PCR primer sets*” for genetic testing of canine plucked hairs or degraded remains (45). During this research each marker has been proved to be an effective tool for investigating population substructure in dogs.

Regarding genetic diversity, we did not find any significant difference between the family and kenneled beagle stocks in allele frequencies, observed heterozygosities, and inbreeding coefficients. This latter was rather surprising as all our kenneled dogs could be traced back to the breeding stock of one commercial breeder. Moreover, the inbreeding coefficient (F_IS_) was 0.053 for the whole analyzed beagle population which does not exceed the critical value of 12.5% (54), thus represents no significant level of inbreeding. Comparing the obtained F_IS_ to other breeds in Hungary, analyzed by a partially overlapping marker set, our results were similar to those of Dachshunds but somewhat smaller than in German shepherds or Giant Schnauzers (36). As to date there is no published STR data available from Hungarian beagles, or from world-wide populations, we compared our F_IS_ data to a Polish beagle dataset that was collected from the Cracow area, and inbreeding was calculated based on pedigree documents (79). In this population the mean inbreeding coefficient was 0.007 for the overall population and 0.049 for inbred animals, lower than the inbreeding coefficient in our study (0.053).

To test the genetic divergence between the kenneled and family stocks we calculated the genetic substructure in the population by measuring the proportion of genetic variance of the eleven microsatellite loci that can be attributed to the variance between the stocks (F_ST_, R_ST_). When calculating all loci together there was no significant differences detected between the two stocks by conventional F-statistics or by using AMOVA. However, when calculating genetic substructure on each locus separately, we found significant F_ST_ and R_ST_ values at seven and two loci, respectively. These observed small discrepancies could be due to sampling effect because of the few genotypes involved into the study, especially in case of the family dogs (only to 27 animals). Unfortunately there are only limited data available of the inter-population variances within any dog breeds in Hungary, or for the beagle breed itself. Our study is the first intra-breed genetic comparison using microsatellites in this breed. Thus, we can only indirectly compare our results to those of previous studies. For example, when analyzing the genetic divergence between mixed breed and purebred dogs on a Hungarian dog population (36), the calculated F_ST_ values in the pooled samples varied between 0.042 and 0.158 at the ten STR loci involved, and all represented significant difference. Similar values were observed when analyzing the molecular variance (Φ_ST_), and the majority of these were also significant. The same level of genetic divergence was observed between different breeds (German shepherd, beagle and greyhound) in a South Korean population (80). Compared to these, the overall F_ST_ and R_ST_ values in our beagle population were below 1% (0.001 and 0.008, respectively), which are also much lower than observed in human populations (81). The weak signal of population structure was confirmed by the Structure analysis. The model could not separate the family and kenneled individuals into different clusters even if population subdivision was added in the model. Only when information about the location of sampling was also added to the model did we find any overlap with the dogs' origin and keeping conditions. In this model version, the majority of the family dogs were assigned into a separate cluster, while the kenneled dogs still shared both clusters. However, we need to note that the majority of family dogs were kept by different owners, so grouping these individuals into one location group may have biased these results. Thus, based on the population genetic analyses, we can conclude that the inter-population variance found at multi-locus level in our sample does not represent a significant genetic divergence between kenneled and family beagle stocks, thus differences in the genetic structure do not explain the different behavior of family and kenneled dogs on the population level.

When interpreting these results, certain limitations of our study need to be noted. First, the low sample size, particularly in the adopted and family groups, could affect the reliability of the results, especially for the population-genetic analyses. Second, the genetic analyses in the current study are aimed only at ruling out the possibility of population-level genetic divergence behind the observed behavioral differences, they are inadequate to address any questions regarding the possible genetic background of the phenotype itself. Further genome-wide association studies targeting this particular behavior are needed to explore its genetic basis and identify possible candidate regions.

## Summary and Conclusions

Taken together, the strong difference in Responsiveness between pet and kenneled dogs confirms the general negative effect of the restricted environment and limited experiences on the dogs' behavior. The high responsiveness of the adopted dogs further emphasizes the role of the environment, socialization and life experiences in the regulation of this behavior. However, the effect of the environment alone could not fully explain our results. We found a large behavioral variability within the uniformly kept and socialized kenneled dogs (reflecting a general difference in their stress handling), which implies that the role of genetic factors. Although, we found no difference in the genetic diversity, nor any evidence of significant genetic divergence between the family and kenneled stocks, these do not exclude the possibility of a genetic basis behind the observed behavioral variability, especially within the kenneled stock.

This combination of results provides some support for a gene-environment interaction behind the behavior difference of the pet and kenneled dogs. Some individuals (in the beagle breed) could be genetically predisposed to show strong stress responses (e.g., freezing) for social and environmental challenges, and the restrictive environment acts as a catalyst lowering the threshold when these behaviors activate. Dogs living in a typical family environment could also be genetically sensitive to stress but since these dogs are not subjected to social and environmental deprivation, their genetic sensitivity does not manifest in normal circumstances. Further genetic analyses of the two extremes of kenneled dogs (i.e., unresponsive and responsive) may reveal more about the genetic factors behind this predisposition, and may allow a greater understanding of the genetics behind stress- and fear-related behaviors in general. Moreover, the fact that 23% of the kenneled dogs displayed behaviors similar to family dogs confirms that some animals are genetically less sensitive to the stress caused by novel stimuli and human handling, and also that this higher tolerance can be a target of selection. Therefore, identifying these animals could also help to improve the general welfare of kenneled dogs, as future generations could be selectively bred for higher resilience to the stress caused by standard experimental procedures.

## Data Availability Statement

The datasets generated for this study can be found in the article/Supplementary Material.

## Ethics Statement

A written statement (PE/EA/3742-4/2016) was obtained from the Food Chain Safety and Animal Health Directorate Government Office based on the decision of the Scientific Ethic Council of Animal Experiments. According to this statement and the corresponding definition by law, the current non-invasive observational study is not an animal experiment, therefore it is currently allowed without need for permission from the University Institutional Animal Care and Use Committee (UIACUC, Eötvös Loránd University, Hungary).

## Author Contributions

EK, BT, JT, and BE contributed to the conception and design of the study. JT and EK were responsible for funding acquisition. The recruitment of participants was largely achieved by LB. BT, EK, and EP carried out the behavior experiments, while KT and BE carried out the genotyping and molecular analyses. BT performed the statistical analyses for the behavioral, and KT and BE for the genetic data. BT and KT wrote the first draft of the manuscript. All authors contributed to manuscript revision, and read and approved the submitted version.

### Conflict of Interest

The authors declare that the research was conducted in the absence of any commercial or financial relationships that could be construed as a potential conflict of interest.
