# Supplementary material for: Comparison of Behavior and Genetic Structure in Populations of Family and Kenneled Beagles
Source: Front Vet Sci. 2020 Apr 15;7:183. doi: 10.3389/fvets.2020.00183 (PMC7174610; doi:10.3389/fvets.2020.00183)

Supplementary Figure 1. Dendrogram illustrating the result of the hierarchical cluster analysis based on the Responsiveness score. The squares represent the individuals, their colors represent their group (dark grey: family dogs; middle grey: adopted dogs; light grey: kenneled dogs). The length of horizontal lines represent the distance between the clusters. The last line joining the two large clusters together is not shown in proportion due to its length.

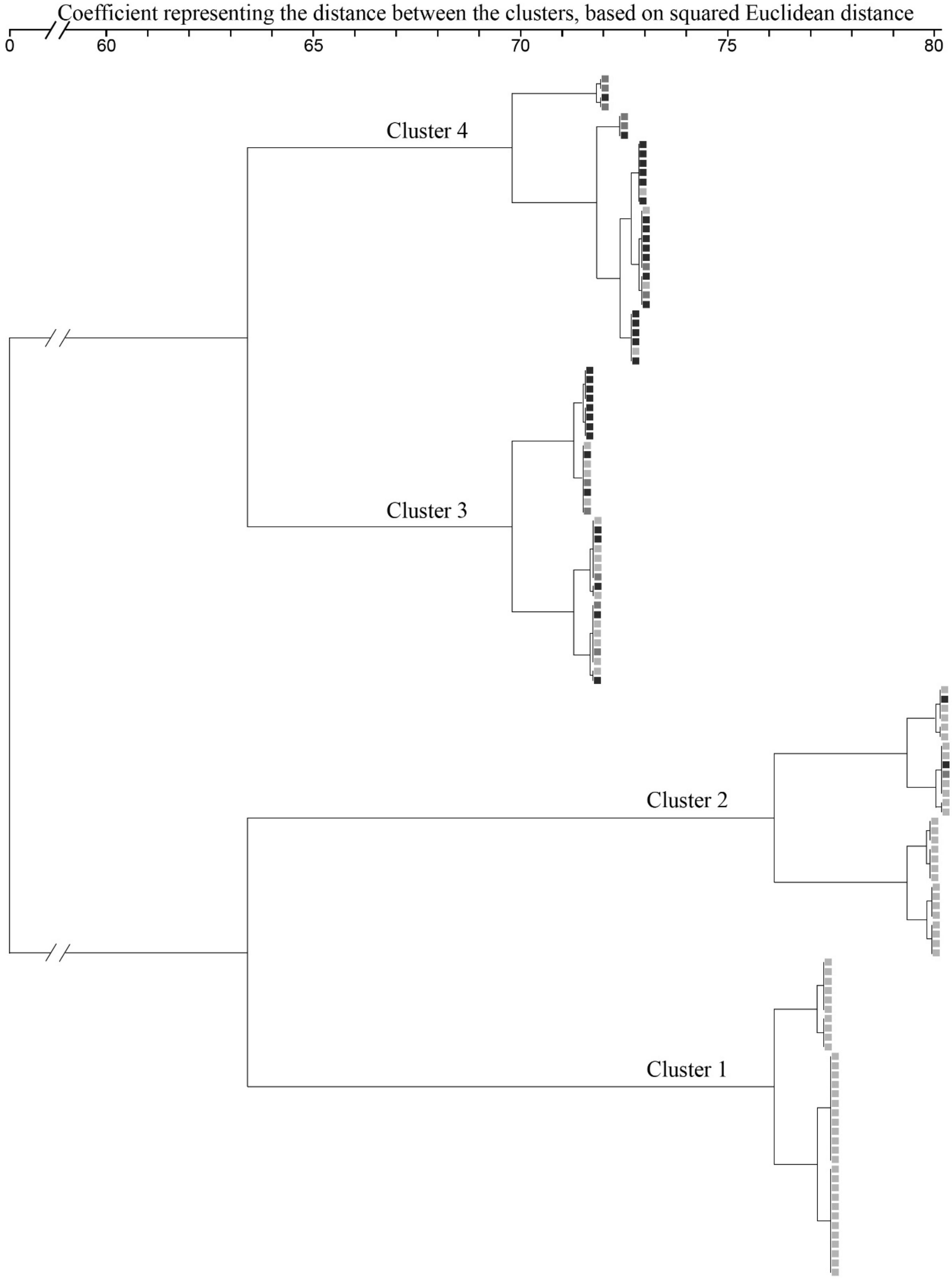

Supplement: Supplementary file 1 [file Image_1.pdf]
