# Supplementary material for: Comparison of Behavior and Genetic Structure in Populations of Family and Kenneled Beagles
Source: Front Vet Sci. 2020 Apr 15;7:183. doi: 10.3389/fvets.2020.00183 (PMC7174610; doi:10.3389/fvets.2020.00183)
Supplement: Supplementary file 2 [file Table_1.docx]

**Supplementary Table 1.** Demographic characteristics and keeping conditions of the family, adopted, and kenneled dogs.

| **Demographic characteristics** | **Family dogs** | **Adopted dogs** | **Kenneled dogs** | |
| --- | --- | --- | --- | --- |
|  |  |  | Institute 1 | Institute 2 |
| N of dogs participating | 37 | 13 (from 5 litters) | 55 | 23 |
| origin of the dogs | breeder: 32 dogs; adopted from other families: 2 dogs; no info: 3 dogs | all bred by Institute 1 | 15 dogs from one commercial breeder, 40 dogs facility-bred | all from one commercial breeder (the same breeder as Institute 1) |
| age (mean± SD) | 3.28 ±2.76 years (range: 1 to 10 years) | 1.10 ±0.09 years (range: 0.9 to 1.3 years) | 2.53 ± 1.01 years (range: 1 to 6 years) | 2.11 ± 1.70 years (range: 1 to 6.6 years) |
| sex ratio | 18 males, 19 females (2 males and 4 females neutered) | 6 males, 7 females (3 males neutered) | 40 males, 15 females (none neutered) | 12 males, 11 females (none neutered) |
| **Keeping conditions** |  |  |  |  |
| age at acquisition | 0-10 weeks: 28 dogs; 3 months-1year: 5 dogs; 2 years: 1 dog; no info: 3 dogs | all dogs at 8 weeks of age | born at the institute: 40 dogs; > 1 year: 15 dogs | all dogs: > 1 year |
| keeping place | only in house: 15 dogs; both in house and garden: 12 dogs; mostly in garden: 7 dogs; no info: 3 dogs | only in house: 1 dog; both in house and garden: 8 dogs; mostly in garden: 4 dogs | indoor kennels with permanent access to outdoor runs | indoor kennels, no access to outdoor runs |
| other dogs in the household | none: 17 dogs; 1: 13 dogs; 2 or more: 4 dogs; no info: 3 dogs | none: 7 dogs; 1: 6 dogs | 10-15 dogs living in one kennel | same-sex pairs in one kennels, all kennels in the same room |
