# Supplementary material for: Comparison of Behavior and Genetic Structure in Populations of Family and Kenneled Beagles
Source: Front Vet Sci. 2020 Apr 15;7:183. doi: 10.3389/fvets.2020.00183 (PMC7174610; doi:10.3389/fvets.2020.00183)
Supplement: Supplementary file 4 [file Table_3.docx]

**Supplementary Table 3.** Locus name, localization, amplified fragment length of alleles, repeat structure and motif, fluorescent dye, PCR primer sequences and concentrations applied for the 11 examined DNA markers. CFA: *Canis familiaris* Autosome, CFX: *Canis familiaris* X chromosome, F: Forward primer sequence, R: Reverse primer sequence. The markers examined are constructed of at least 3, maximum 6 nucleotide length repeats, and both simple and compound repeat motifs can be observed among the markers. Inter-allelic variants have been described at 3 loci – FH2054, FH2584, WILMS-TF – during the study).

| **Locus** | **Chromosome** | **Allele size range (bp)** | **Repeat structure** | **MiniPlex** | **Dye** | **Forward/Reverse PCR primers** | **Primer conc (nM)** | **Reference** |
| --- | --- | --- | --- | --- | --- | --- | --- | --- |
| PEZ1 | CFA7 | 98-122 | TACA  Simple | I | 6-FAM | F: GGCTGTCACTTTTCCCTTTC  R: CACCACAATCTCTCTCATAAATAC | 4.1 | Primmer and Matthews, 1993 |
| PEZ5 | CFA123 | 130-151 | TTTA  Simple | I | VIC | F: GCTATCTTGTTTCCCACAGC  R: GTCACTGTATACAACATTGTC | 1.1 | Halverson and Basten, 2005 |
| PEZ3 | CFA19 | 122-140 | GAA  Compound | I | NED | F: CACTTCTCATACCCAGACTC  R: CAATATGTCAACTATACTTC | 5.3 | Halverson and Basten, 2005 |
| PEZ21 | CFA2 | 94-110 | AAAT  Simple | I | PET | F: AACCGGTTGTGATTTCTGGG  R: GTCTGTGTCATTAGTGACATC | 3.5 | Halverson and Basten, 2005 |
| PEZ16 | CFA27 | 158-190 | GAAA or GGAA  Compound | I | 6-FAM | F: GCTCTTTGTAAAATGACCTG  R: ATCAGGGCAGTTTGGCACACT | 1.8 | DeNise et al., 2004 |
| REN124F09 | CFA3 | 131-146 | TTTTA  Compound | I | VIC | F: CAAGGGTCCCCCATATTGC  R: CTGGCTCTATCTCTCTGTC | 1.1 | Zenke 2010 |
| FH2054 | CFA12 | 142-166 | GATA  Compound | II | 6-FAM | F: GCCTTATTCATTGCAGTTAGGG  R: ATGCTGAGTTTTGAACTTTCCC | 4.3 | Pádár 2006 |
| PEZ19 | CFA20 | 134-146 | AAAT  Simple | II | VIC | F: GACTCATGATGTTGTGTATC  R: TCTCTACTGTCTTGCTCTCT | 4.3 | Zenke 2010 |
| FH2584 | CFX | 148-176 | CTTT  Compound | II | NED | F: TCCCTCTGCTTACTTGCAAA  R: GCAGAAAGTATGTTGCTCCT | 5.3 | Zenke 2010 |
| WILMS-TF | CFA18 | 158-186 | GAAA  Compound | II | PET | F: CCCAATCTCCAGAGATTTTCC  R: CCCACTGTTCTGTGGTTTGC | 3.6 | Zenke 2010 |
| vWF.X | CFA27 | 162-168 | AGGAAT  Simple | II | VIC | F: CTCCCCTTCTCTACCTCCACCTCTAA  R: CAGAGGTCAGCAAGGGTACTATTGTG | 1.1 | Eichmann et al. 2004 |

References cited:

DeNise S, Johnston E, Halverson J, Marshall K, Rosenfeld D, McKenna S, et al. Power of exclusion for parentage verification and probability of match for identity in American kennel club breeds using 17 canine microsatellite markers. Anim Genet. 2004;35(1):14–17.

Eichmann C, Berger B, Parson W. A Proposed Nomenclature for 15 Canine Specific Polymorphic Canine STR Loci for Forensic Purposes. Int J Legal Med. 2004;118:249-66.

Halverson J, Basten C. A PCR Multiplex and Database for Forensic DNA Identification of Dogs. J Forensic Sci. 2005;50(2):JFS2004207-12.

Pádár Zs. Kutya Eredetű Anyagmaradványok Igazságügyi Genetikai Vizsgálata. Doctoral thesis, Szent István University; 2006.

Primmer CR, Matthews ME. Canine tetranucleotide repeat polymorphism at the VIAS-D10 locus. Anim Genet. 1993;24(4):332-332.

Zenke, P. Mikroszatellita-Polimorfizmusok Vizsgálata Kutya Eredetű Anyagmaradványokból. Doctoral thesis, Szent István University; 2010.
