# Supplementary material for: Comparison of Behavior and Genetic Structure in Populations of Family and Kenneled Beagles
Source: Front Vet Sci. 2020 Apr 15;7:183. doi: 10.3389/fvets.2020.00183 (PMC7174610; doi:10.3389/fvets.2020.00183)
Supplement: Supplementary file 5 [file Table_4.docx]

**Supplementary Table 4.** Comparison of sex ratio, age, and ratio of the subgroups between the four clusters, separately for family, adopted, and kenneled dogs. For the family and adopted dogs, only cluster1 and 2 were compared. For kenneled dogs, the four dogs in cluster 1 were merged with those in cluster 2 for the statistical comparisons.

|  |  | **Cluster 1** | **Cluster 2** | **Cluster 3** | **Cluster 4** | **statistics** |
| --- | --- | --- | --- | --- | --- | --- |
| Family dogs | | | | | | |
|  | N | 0 | 2 | 15 | 20 | - |
|  | sex | - | 50% male | 40% male | 55% male | χ^2^: 0.772; p = 0.380 |
|  | age (mean ±SD) | - | 5.05 ±1.35yr | 4.19 ±3.26yr | 2.73 ±2.35yr | z = 1.645; p = 0.104 |
|  | subgroups | - | 1 tested with E | 27% tested with E | 40% tested with E | χ^2^: 0.676; p = 0.411 |
| Adopted dogs | | | | | | |
|  | N | 0 | 1 | 5 | 7 | - |
|  | sex | - | female | 40% male | 57.1% male | χ^2^: 0.343; p = 0.558 |
|  | age (mean ±SD) | - | 1.10yr | 1.07 ±0.03yr | 1.11 ±0.11yr | z = 0.081; p = 0.935 |
| Kenneled dogs | | | | | | |
|  | N | 34 | 26 | 14+4 | | - |
|  | sex | 76.5% male | 61.5% male | 55.6% male | | χ^2^: 2.778; p = 0.249 |
|  | age (mean ±SD) | 2.18 ±0.81yr | 2.57 ±1.49yr | 2.77 ±1.6yr | | z = 1.089; p = 0.580 |
|  | subgroups | 70.6% Institute 1 | 61.5% Institute 1 | 83.3% Institute 1 | | χ^2^: 2.430; p = 0.297 |
